# Kernel-Weighted Aggregation for Hyperparameter-Free Quantum Federated Learning

**DOI:** 10.3390/e28090958

**Published:** 2026-08-26

**Authors:** Rui Huang

**Affiliations:** School of Artificial Intelligence, Shenzhen Polytechnic University, Shenzhen 518055, China; huangrui@szpu.edu.cn

**Keywords:** quantum federated learning, kernel density estimation, non-IID data, variational quantum circuits, aggregation methods

## Abstract

Quantum federated learning (QFL) enables collaborative training of variational quantum circuits across decentralized clients, but client drift under non-IID data distributions degrades standard Federated Averaging (FedAvg). Existing aggregation methods either introduce tunable hyperparameters that depend on unknown data heterogeneity or incur structural costs such as order-dependent error propagation. We propose Kernel-Weighted Aggregation (KWA), a hyperparameter-free aggregation strategy that constructs a non-parametric kernel density estimate over the received client parameter vectors at each communication round. Clients in high-density regions receive greater voting power, and isolated clients are automatically attenuated. The kernel bandwidth is the median of all pairwise squared distances. We evaluate KWA against six state-of-the-art QFL methods and five classical robust baselines on five binary MNIST digit-pair tasks under IID, Dir(0.5), and Dir(0.1) distributions with a unified four-qubit benchmark. Under IID data, KWA recovers the performance of FedAvg. Under Dir(0.5), KWA attains the highest average accuracy (0.7120), followed by FedAvg (0.7093). The margin at N=4 clients is not statistically significant. It grows to +0.023 at N=16 in the client scaling experiment. Under this distribution, the five classical robust baselines also rank below FedAvg. Under Dir(0.1), KWA exceeds FedAvg by 0.028 on average, with a pooled paired *t*-test p=0.068. Larger-circuit and three-class experiments show that the advantage does not yet transfer consistently beyond the four-qubit binary setting. KWA thus provides a hyperparameter-free aggregation strategy that recovers FedAvg under IID conditions, adapts to client drift, and requires no manually tuned hyperparameters.

## 1. Introduction

Quantum computing has progressed from a theoretical proposition [1] to the Noisy Intermediate-Scale Quantum (NISQ) era [2]. Near-term quantum processors with tens to hundreds of imperfect qubits can execute algorithms beyond the reach of classical simulation. Variational quantum circuits (VQCs) [3] have emerged as a leading paradigm for NISQ computation. A parameterized quantum circuit is optimized by a classical optimizer to minimize a problem-specific cost function. The quantum device serves as an efficient evaluator of the loss landscape. This hybrid quantum-classical workflow has been applied to classification [4,5,6], regression [7], generative modeling [8], combinatorial optimization [9], and meta-learning [10]. It constitutes the backbone of the rapidly expanding field of quantum machine learning (QML) [11,12,13].

A central practical challenge for applying QML at scale is data availability. In many real-world settings, training data are distributed across multiple organizations or devices and cannot be centralized, owing to privacy regulations, bandwidth constraints, or proprietary concerns. Federated learning (FL) [14,15] addresses this constraint by keeping data local. Each client trains a model on its private dataset and only shares model parameters with a central server. The server aggregates the contributions into a global model. Federated Averaging (FedAvg) is the canonical aggregation algorithm, computing an unweighted mean of the received parameter vectors. FL has been successfully applied in classical machine learning across domains including mobile keyboard prediction, healthcare, and finance [16,17].

Quantum federated learning (QFL) extends the FL paradigm to VQCs: clients train local VQC models and the server aggregates their parameters. The first QFL framework was established by Huang et al. [18], who demonstrated that a star-topology QFL system with FedAvg aggregation achieves classification accuracy comparable to centralized training on the MNIST dataset. Subsequent studies have explored blind quantum computing protocols for secure aggregation [19], quantum error mitigation in federated settings [20], and the extension of QFL to multi-class and high-dimensional tasks [21]. A recent survey provides a systematic account of QFL foundations and challenges [22]. However, the core aggregation step in nearly all QFL studies to date remains FedAvg: the server computes the arithmetic mean of the received parameter vectors, assigning equal weight 1/N to each client.

Equal weighting is optimal when client data are Independently and Identically Distributed (IID), because all local models converge to similar regions of parameter space. Under non-Independently and Identically Distributed (non-IID) data, however, local models drift toward distinct, client-specific optima during local training [23]. A single divergent client can pull the global model away from the population consensus with the same voting power as every other client. In classical FL, proximal regularization [24] and control variates [25] mitigate drift at the local training step. The present work focuses on the server-side aggregation step. Client drift has motivated alternative QFL aggregation strategies that depart from uniform weighting. The Weighted and Personalized methods of Gurung et al. [26] blend local models with the global average through fixed interpolation coefficients or apply a hard distance-based decision rule. The Chain method [27] arranges clients in a sequential training pipeline, eliminating the central server but introducing order-dependent error propagation. The Clustered method [28] partitions parameter vectors via *K*-means and averages cluster representatives, requiring a pre-specified number of clusters *K*. The Density method [29] weights clients by their likelihood under a Gaussian Mixture Model (GMM) fit to the parameter vectors, requiring the number of GMM components $K_{\mathrm{GMM}}$. Each of these methods was validated under a distinct experimental configuration, with different VQC architectures, qubit counts, optimizers, and random seeds, and each reported advantages over FedAvg. Their claims, however, have not been tested under a controlled, unified comparison.

Two structural patterns emerge from the methods discussed above. First, methods that combat drift through hyperparameters shift the burden to the practitioner. The Weighted, Clustered, and Density methods require $\alpha_{g}$ and $\alpha_{\ell }$, *K*, and $K_{\mathrm{GMM}}$, respectively. These values must be selected without knowledge of the true data distribution at training time. Second, methods that avoid hyperparameters incur alternative costs. Personalized outputs per-client models rather than a global model. Chain introduces order sensitivity. A hyperparameter-free aggregation strategy that adaptively weights clients based on the observed parameter geometry without requiring manual configuration would address both limitations simultaneously.

In this work, we propose Kernel-Weighted Aggregation (KWA), a hyperparameter-free aggregation strategy for non-IID QFL. KWA treats the *N* local parameter vectors received by the server as samples from an unknown distribution in $R^{d}$ and constructs a non-parametric kernel density estimate. The centrality of each client, defined as the sum of its Gaussian kernel affinities to all other clients, determines its aggregation weight. Clients in high-density regions receive greater voting power; isolated outliers are automatically attenuated. The kernel bandwidth is set to the median of all pairwise squared distances between parameter vectors, a purely data-driven heuristic that adapts round by round and introduces no tunable hyperparameters. Under IID conditions, KWA reduces to standard FedAvg as the pairwise distances approach zero. We evaluate KWA against the six state-of-the-art QFL aggregation methods described above and five classical robust baselines, twelve methods in total, on five binary MNIST digit-pair classification tasks under IID and Dir(0.5) non-IID data distributions in the main benchmark. A stronger-heterogeneity benchmark uses Dir(0.1). All methods are re-implemented within a unified benchmark using the same VQC architecture (ZZFeatureMap with circular entanglement, q=4 qubits, d=8 parameters), the same optimizer (Adam, learning rate 0.1), the same training protocol (T=10 rounds, S=30 local steps), and the same five random seeds. We also test a larger circuit with q=6 qubits and a three-class MNIST task. Ablation studies cover the bandwidth selection heuristic, the kernel function, and the scaling behavior of KWA with the number of clients (N∈{4,8,16}).

The remainder of this paper is organized as follows. Section 2 reviews the QFL framework and the six existing aggregation methods evaluated in this work. Section 3 derives the KWA procedure, analyzes its theoretical properties, and presents the algorithm in pseudocode. Section 4 reports the benchmark results under IID, Dir(0.5), and Dir(0.1) distributions, the convergence and weight evolution of KWA, and the client scaling trend. Section 5 discusses the implications of hyperparameter-free aggregation, the limitations of the present research, and directions for future work. Appendix A, Appendix B, and Appendix C contain the ablation studies on bandwidth selection, kernel function choice, and client scaling, respectively. Appendix D and Appendix E report the larger-circuit and three-class experiments.

## 2. Background and Related Work

### 2.1. Quantum Federated Learning Framework

A quantum federated learning (QFL) system [18] consists of a parameter server and *N* clients connected by a communication network. Each client k∈{1,…,N} holds a private local dataset $D_{k}={\left\{\left(x_{i},y_{i}\right)\right\}}_{i=1}^{n_{k}}$ of size $n_{k}$, drawn from distribution $P_{k}$. The system objective is to train a shared variational quantum circuit (VQC) classifier f(x;θ) by solving

(1) $$\min_{\theta }\;L\left(\theta \right)=\sum_{k=1}^{N}\frac{n_{k}}{n}\;L_{k}\left(\theta \right),$$

and $n=\sum_{k=1}^{N}n_{k}$, while ensuring that no client reveals its raw data to the server or to other clients.

The VQC comprises two stages: a data encoding feature map $U_{\phi }\left(x\right)$, which maps classical inputs to quantum states, and a parameterized ansatz U(θ), which performs the classification. We adopt the ZZFeatureMap [4] with circular entanglement as the encoding circuit. The ansatz consists of alternating layers of single-qubit $R_{Y}\left(\theta_{i}\right)$ and $R_{Z}\left(\theta_{j}\right)$ rotations interleaved with nearest-neighbor CNOT gates, forming a hardware-efficient structure. The binary prediction is obtained from the expectation value $\langle Z_{0}\rangle \in \left[-1,1\right]$ measured on the first qubit after executing the full circuit. With q=4 qubits, the circuit contains d=8 trainable parameters. Input data are preprocessed to four dimensions via PCA followed by MinMax scaling before encoding.

Training proceeds in synchronous communication rounds t=0,…,T−1. At the start of round *t*, the server broadcasts the current global model $\theta^{\left(t\right)}$ to all clients. Each client *k* initializes $\theta_{k}^{\left(t\right)}\leftarrow \theta^{\left(t\right)}$, then performs *S* local stochastic gradient steps using the Adam optimizer [30] to minimize the mean squared error loss:

(2) $$L_{k}\left(\theta \right)=\frac{1}{n_{k}}\sum_{i=1}^{n_{k}}{\left(f\left(x_{i};\theta \right)-y_{i}\right)}^{2}.$$

The resulting local parameters $\theta_{k}^{\left(t+1\right)}$ are uploaded to the server, which applies an aggregation function A to produce the next global model:

(3) $$\theta^{\left(t+1\right)}=A\left({\left\{\theta_{k}^{\left(t+1\right)}\right\}}_{k=1}^{N}\right).$$

The canonical choice for A is Federated Averaging (FedAvg) [14], defined as the unweighted arithmetic mean:

(4) $$A_{\mathrm{FedAvg}}\left(\left\{\theta_{k}\right\}\right)=\frac{1}{N}\sum_{k=1}^{N}\theta_{k}.$$

The local-training and global-aggregation cycle repeats for *T* rounds.

Data heterogeneity across clients constitutes the primary challenge in QFL. If the local distributions $P_{k}$ differ substantially, local models drift toward distinct, client-specific optima during the *S* local steps. The aggregated global model may then fail to represent any individual client’s objective. Following the protocol of prior QFL studies [26,27], we simulate heterogeneous data partitions via a symmetric Dirichlet distribution Dir(α). The concentration parameter α governs non-IID severity: α→0 yields extreme label imbalance where each client holds samples predominantly from a single class; α→∞ approaches uniform IID splits. Our experiments cover the IID regime and moderate non-IID conditions at α=0.5.

### 2.2. Existing Aggregation Methods

Client drift under non-IID data has motivated several aggregation strategies that depart from uniform averaging. We review the six methods evaluated in this work. Table 1 summarizes their key properties.

FedAvg (baseline): Huang et al. [18] established the first QFL framework and adopted standard FedAvg as the aggregation mechanism. The method assigns equal weight 1/N to every client regardless of local data distribution or model quality. This uniform treatment is optimal under IID data but becomes a liability under non-IID conditions: a single divergent client contributes equally to the aggregate, shifting the global model toward the outlier in proportion to its weight 1/N. FedAvg requires no manually tuned hyperparameters and serves as the reference baseline throughout this work.

wpQFL (Weighted and Personalized): Gurung et al. [26] introduced two strategies under the wpQFL framework. The Weighted variant computes a per-client blended model $\theta_{k}^{\prime }=\left(\alpha_{g}\theta_{g}+\alpha_{\ell }\theta_{k}\right)/\left(\alpha_{g}+\alpha_{\ell }\right)$, linearly interpolating between the global average and each local model. The fixed coefficients $\alpha_{g}$ and $\alpha_{\ell }$ dampen but cannot eliminate outlier influence. The Personalized variant applies a hard decision rule. Each client computes two squared Euclidean distances: $d_{g}={∥\theta_{k}-\theta_{g}∥}^{2}$ to the global model and $d_{\ell }={∥\theta_{k}-\theta_{k}^{\mathrm{prev}}∥}^{2}$ to its own previous parameters. If $d_{g}<d_{\ell }$, the client retains $\theta_{g}$; otherwise it adopts the midpoint $(\theta_{g}+\theta_{k})/2$. Both approaches output per-client models rather than a single global model, which complicates server-side operation. The Personalized variant additionally stores previous-round parameters, incurring memory overhead linear in *N*.

ccQFL (Chain): Gurung et al. [27] eliminated the central server by arranging clients in a sequential training chain. Each client receives the previous client’s trained model, performs local optimization, and forwards the result to the next client. The last client’s parameters become the global model. This decentralized design avoids the single point of failure inherent in star topologies. However, the chain structure introduces order dependency: the global model is determined entirely by the final client’s local data and training dynamics. If early clients hold heavily skewed data, their errors propagate and compound through the chain. The sequential communication pattern also incurs latency linear in *N*.

mdQFL (Clustered): Gurung et al. [28] proposed a clustering-based strategy. The method partitions the received parameter vectors into *K* groups via *K*-means, selects the member closest to each cluster centroid as the representative, and returns the average of representatives. Clustering discards extreme outliers by isolating them in low-cardinality groups, thereby reducing their influence on the global model. However, the number of clusters *K* is a free hyperparameter, and *K*-means introduces non-determinism through random centroid initialization. With only N=4 clients, the method operates near its degenerate limit, where clustering reduces to simple outlier removal.

qFedInf (Density): Zhao [29] adopted density-based weighting inspired by statistical inference. The method fits a Gaussian Mixture Model (GMM) to the set of received parameter vectors, then weights each client proportionally to its GMM likelihood. Clients residing in high-density regions of parameter space are amplified; isolated clients are attenuated. While statistically well-motivated, the approach requires specifying the number of GMM components $K_{\mathrm{GMM}}$, and the Expectation-Maximization optimization adds $O(N\cdot K_{\mathrm{GMM}}\cdot d\cdot I_{\mathrm{EM}})$ computation per round. The likelihood surface may be poorly estimated when Nd is small relative to the parameter dimensionality, as is the case for N=4 and d=8.

Each of the six methods discussed in this section was evaluated under a distinct experimental configuration, employing different datasets, VQC architectures, qubit counts, optimizers, and random seeds. Their reported advantages over FedAvg are therefore not directly comparable. Establishing a unified benchmark with controlled configurations is a prerequisite for fair comparison and constitutes one contribution of this work. Two methods required adaptation for compatibility with the iterative training-aggregation protocol. The Clustered implementation selects representatives by proximity to cluster centroids rather than by the loss-and-gradient-norm criterion used in the original mdQFL [28], for which no reference implementation was available. The Density implementation fits the GMM on the received parameter vectors and uses the resulting likelihoods as training-stage aggregation weights, whereas the original qFedInf protocol fits the GMM on input data and applies density weighting at inference time [29].

These adaptations preserve the core principle of each method, clustering-based outlier suppression for Clustered and density-based weighting for Density, while enabling integration into the standard QFL training loop. Classical federated learning has studied robust parameter aggregation extensively. FedProx [24] adds a proximal term to the local objective. SCAFFOLD [25] corrects drift with control variates. Robust aggregation rules suppress outliers by statistical selection. Krum [31] selects the vector with the smallest sum of distances to its nearest neighbors. Multi-Krum averages the *m* vectors with the smallest Krum scores. Coordinate-wise median and trimmed mean [32] replace averaging with robust per-coordinate statistics. The geometric median minimizes the sum of Euclidean distances to all client vectors. We include these five rules as classical baselines. The settings are b=1 for trimmed mean, f=1 for Krum and Multi-Krum, and m=2 for Multi-Krum. KWA differs from these rules. It assigns continuous, data-driven weights to all clients through a non-parametric kernel density estimator rather than selecting or trimming a discrete subset.

## 3. Methods

### 3.1. Motivation

The aggregation methods presented in Section 2 fall into two categories. Methods in the first category retain uniform weighting and are consequently vulnerable to client drift under non-IID data. FedAvg belongs to this category: every client contributes equally regardless of how far its parameters have diverged. Methods in the second category introduce tunable hyperparameters to combat drift. The Weighted variant dampens outliers through fixed interpolation coefficients $\alpha_{g}$ and $\alpha_{\ell }$. The Clustered and Density methods require specifying *K* and $K_{\mathrm{GMM}}$, hyperparameters whose optimal values vary with the unknown degree of non-IID severity. The Personalized and Chain approaches avoid tunable parameters but incur structural costs: Personalized outputs per-client models rather than a single global model, and Chain introduces order-dependent error propagation through its sequential training topology.

An aggregation strategy should satisfy three desiderata. First, it should be hyperparameter-free, requiring no manually tuned hyperparameters. Second, it should adaptively down-weight outlier clients whose parameters have diverged from the population consensus, and correspondingly amplify clients that converge to similar solutions. Third, under IID data where all clients produce near-identical models, it should recover standard FedAvg as a limiting case. We propose Kernel-Weighted Aggregation (KWA), which fulfills all three criteria by treating the received parameter vectors as samples from a kernel density estimator over $R^{d}$.

### 3.2. Kernel-Weighted Aggregation

Consider the server at communication round *t*. It receives *N* local parameter vectors ${\left\{\theta_{k}\right\}}_{k=1}^{N}$, each of dimensionality *d*, from the clients. We regard these vectors as samples from an unknown distribution in $R^{d}$ and construct a non-parametric estimate of the local density around each point. The core idea is that clients whose parameters lie in high-density regions, indicating convergence toward a shared solution, should receive higher aggregation weights than isolated clients whose parameters occupy low-density tail regions. The KWA operator proceeds in six steps, as follows.

Step 1: Pairwise distances: Compute the squared Euclidean distance between every pair of parameter vectors:

(5) $$d_{ij}^{2}={∥\theta_{i}-\theta_{j}∥}^{2},$$

where j∈{1,…,N}.

Step 2: Bandwidth selection: The Gaussian kernel has a single bandwidth parameter *h* that controls the neighborhood radius. We set $h^{2}$ to the median of all pairwise squared distances:

(6) $$h^{2}=\mathrm{median}\left(\{d_{ij}^{2}:1\leq i<j\leq N\}\right).$$

The median is robust to extreme outliers, unlike the mean. This bandwidth is purely data-driven and introduces no tunable hyperparameter. The median rule follows data-driven bandwidth selection for kernel density estimation [33,34].

Step 3: Kernel affinity: For each ordered pair (i,j) with i≠j, compute the Gaussian kernel affinity:

(7) $$a_{ij}=\exp\;\left(-\frac{d_{ij}^{2}}{2h^{2}}\right).$$

For pairs whose distance is small relative to *h*, the affinity approaches 1; for distant pairs, $a_{ij}$ decays toward 0. The Gaussian kernel yields smooth, differentiable weights. Alternative kernel functions, including Laplace and Cauchy, are evaluated in Appendix B; the choice of kernel has negligible impact on classification accuracy.

Step 4: Centrality: Define the centrality $c_{i}$ of client *i* as the sum of its affinities to all other clients:

(8) $$c_{i}=\sum_{j=1,j\neq i}^{N}a_{ij}.$$

A client situated near several others accumulates a high centrality score. An isolated client receives near-zero centrality because its affinities to all peers are negligible. Centrality thus serves as a non-parametric kernel density estimate of the client *i* within the current parameter population.

Step 5: Weight normalization: Normalize the centrality scores to obtain aggregation weights:

(9) $$w_{i}=\frac{c_{i}}{\sum_{k=1}^{N}c_{k}}.$$

The weights are non-negative and sum to one. If the sum of centralities is zero, meaning that all mutual distances are large relative to *h*, we fall back to uniform weights $w_{i}=1/N$.

Step 6: Weighted aggregation: The global model is the weighted average of all local parameter vectors:

(10) $$\theta_{\mathrm{KWA}}=\sum_{k=1}^{N}w_{k}\;\theta_{k},$$

which is the final output of KWA and replaces $A_{\mathrm{FedAvg}}$ in the QFL training loop described by Equation (4).

**Figure 1:**
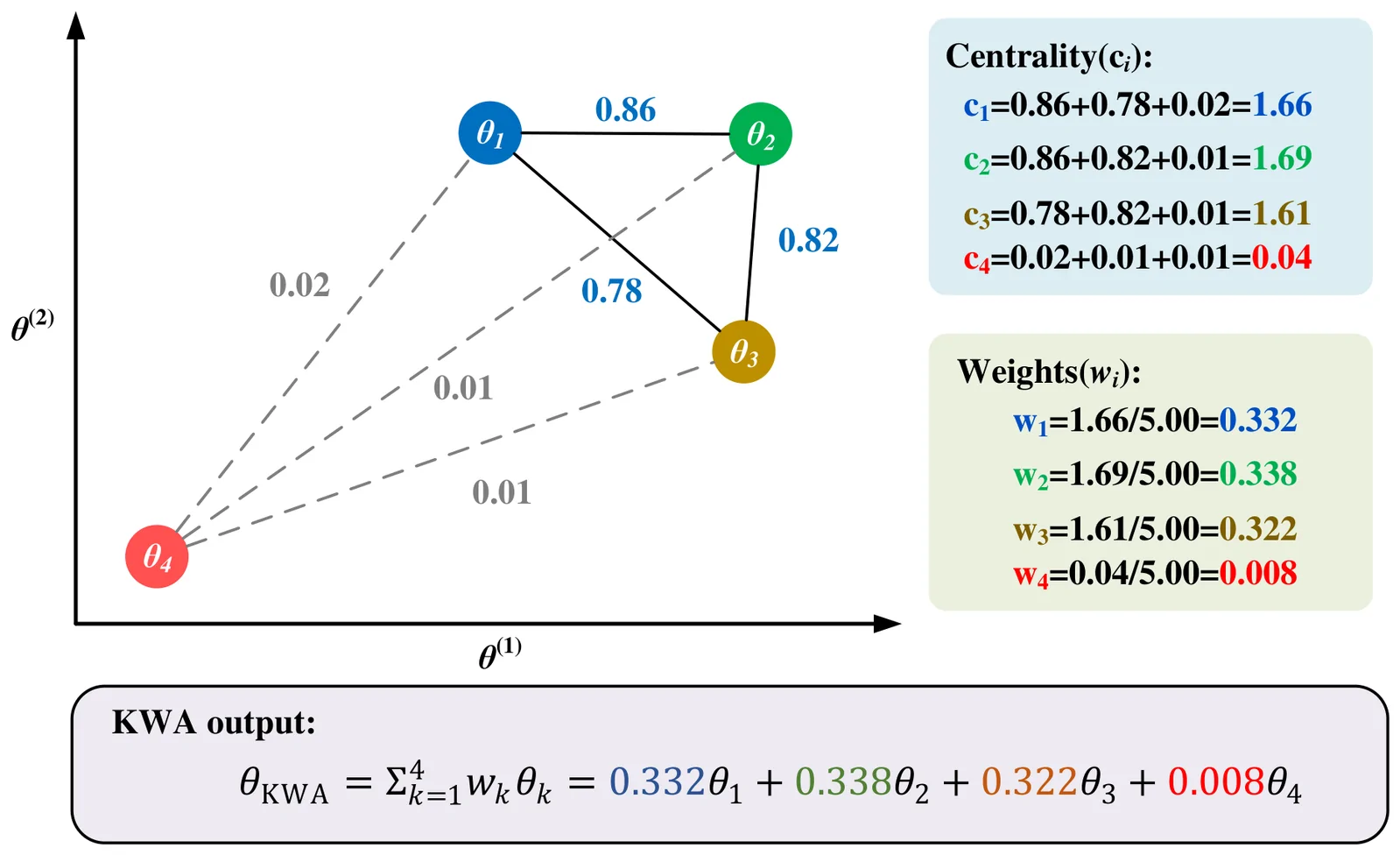
Schematic of Kernel-Weighted Aggregation for N=4 clients. Edges represent pairwise kernel affinities $a_{ij}$ with thickness proportional to affinity strength. Centrality $c_{i}$ is the sum of incident affinities. The isolated client receives near-zero weight; the three mutually close clients dominate the aggregation.

Figure 1 illustrates the KWA mechanism in a two-dimensional parameter space. Four client parameter vectors are shown with their pairwise affinities and resulting centrality-based weights. The isolated client receives a small weight while the three mutually close clients share most of the voting power.

### 3.3. Analysis of KWA

We examine four properties of KWA that characterize its behavior under both IID and non-IID conditions. Proposition 1 collects three elementary properties used throughout the analysis.

**Proposition 1** (Elementary properties of the KWA operator)**.**
*Let ${\left\{\theta_{k}\right\}}_{k=1}^{N}\subset R^{d}$ be the parameter vectors received by the server, let $\theta_{\mathrm{KWA}}$ be the output of Algorithm 1, and let $\bar{\theta }=A_{\mathrm{FedAvg}}\left(\left\{\theta_{k}\right\}\right)=\frac{1}{N}\sum_{k=1}^{N}\theta_{k}$. The following statements hold.*

1. *The output is a convex combination of the received vectors. The weights satisfy $w_{k}\in \left[0,1\right]$ and $\sum_{k=1}^{N}w_{k}=1$*.
2. *If all pairwise squared distances vanish, then $\theta_{\mathrm{KWA}}=\bar{\theta }$*.
3. *The deviation from FedAvg is bounded by the largest client deviation:*
(11) $$∥\theta_{\mathrm{KWA}}-\bar{\theta }∥\;\leq \max_{1\leq k\leq N}∥\theta_{k}-\bar{\theta }∥.$$

**Proof.**  Part (i) follows from the construction in Algorithm 1. Each centrality is a finite sum of Gaussian kernel values, each of which is non-negative, and the weights are the normalized centralities. The fallback branches assign uniform weights. In every branch, the weights are non-negative and sum to one. Part (ii) follows from the bandwidth threshold: if all pairwise squared distances vanish, the median bandwidth is zero and Algorithm 1 returns $\bar{\theta }$. For part (iii), the identity $\sum_{k=1}^{N}w_{k}\left(\theta_{k}-\bar{\theta }\right)=\theta_{\mathrm{KWA}}-\bar{\theta }$ holds because the weights sum to one. The triangle inequality gives

$$∥\theta_{\mathrm{KWA}}-\bar{\theta }∥\;\leq \sum_{k=1}^{N}w_{k}∥\theta_{k}-\bar{\theta }∥\;\leq \max_{1\leq k\leq N}∥\theta_{k}-\bar{\theta }∥.$$

   □

**Algorithm 1:** Kernel-Weighted Aggregation (KWA)

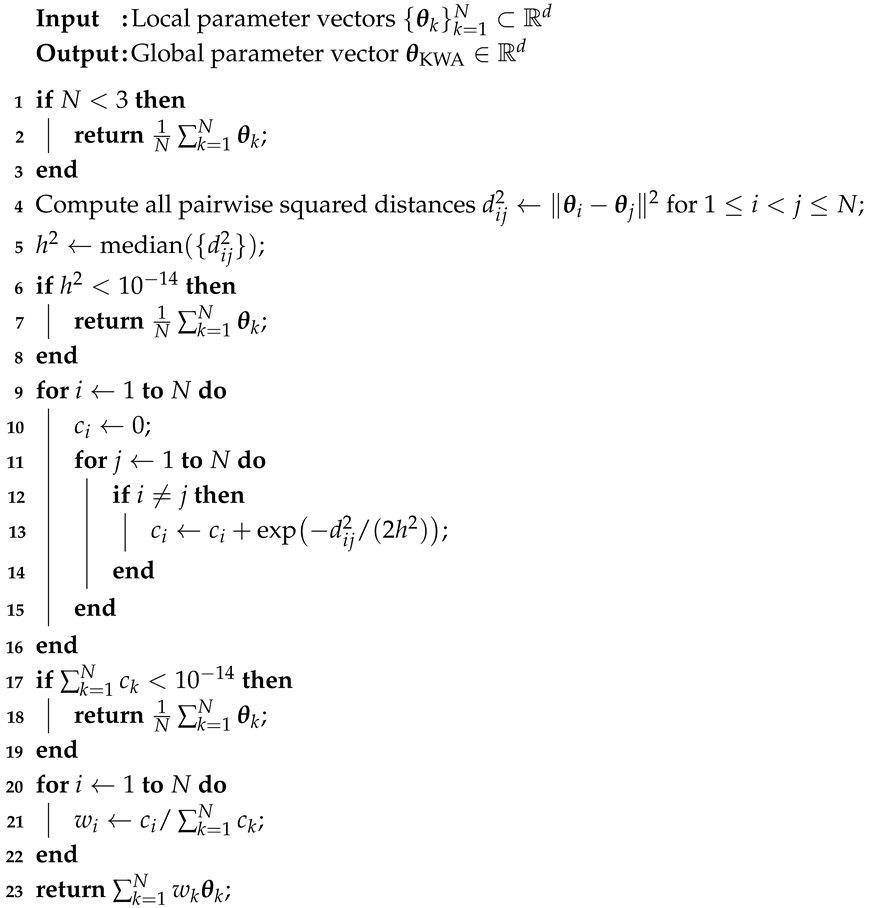



Reduction to FedAvg under IID data: Under IID data, local models converge to similar regions of parameter space after local training. The pairwise distances satisfy $d_{ij}^{2}\approx 0$ for all i,j, yielding $a_{ij}\approx 1$, centrality $c_{i}\approx N-1$ for all *i*, and consequently $w_{i}\approx 1/N$. KWA recovers standard FedAvg in this limit, satisfying the third design desideratum.

Suppression of client drift under statistical heterogeneity: Under non-IID data, a client whose local distribution differs sharply from the others will drift toward a different optimum during local training. The squared distances from this outlier to all other clients become large relative to $h^{2}$, driving the corresponding affinities toward zero. The outlier’s centrality collapses and its aggregation weight approaches zero. This suppression is automatic: KWA requires no hard threshold, no pre-specified outlier definition, and no sensitivity tuning. The property concerns honest clients whose local distributions differ. KWA is not designed for Byzantine adversaries. The benefit of this adaptive mechanism grows with the number of clients. Appendix C reports a scaling experiment where the KWA advantage over FedAvg widens from +0.0005 at N=4 to +0.023 at N=16 under non-IID data, confirming that centrality-based weighting becomes increasingly valuable as the federation expands.

The data-driven bandwidth $h^{2}$ enables a second adaptive mechanism. In rounds where client parameters are tightly clustered, $h^{2}$ is small and the kernel sharpens, amplifying even modest differences in parameter quality. In rounds with large dispersion, $h^{2}$ grows and the kernel flattens, preventing premature commitment to any single region of parameter space. This round-by-round adaptation mirrors the natural progression of QFL training: early rounds exhibit large parameter scatter as clients explore their local landscapes, while later rounds show convergence toward narrower basins.

Hyperparameter-free operation: KWA has no tunable hyperparameters. The only free quantity is the bandwidth $h^{2}$, determined entirely from the data at each round via the median heuristic of Equation (6). This contrasts with Weighted ($\alpha_{g},\alpha_{\ell }$), Clustered (*K*), and Density ($K_{\mathrm{GMM}}$), all of which demand hyperparameter choices that must be set without knowledge of the operating conditions. We distinguish between a modeling choice and a hyperparameter. A modeling choice selects the functional form of an operation: the Gaussian kernel, the Euclidean distance metric, and the median aggregator are fixed design decisions, not quantities to be tuned. A hyperparameter is a free scalar or integer that must be assigned a value before the method can execute. KWA involves only modeling choices: the median heuristic produces a bandwidth from the data deterministically, and there is no free value for the practitioner to specify. The ablation results support this distinction. Replacing the median with the mean changes the average accuracy by 0.001 under IID and by 0.0045 under Dir(0.5) (Appendix A), and replacing the Gaussian kernel with Laplace or Cauchy changes performance by less than 0.004 (Appendix B). KWA is therefore insensitive to the specific modeling choices it makes.

Computational complexity: KWA requires $O(N^{2}d)$ floating-point operations per round for computing pairwise distances and affinities. For N=4 and d=8, this amounts to 6 distance computations and 12 kernel evaluations, negligible alongside the VQC cost of executing S·N training steps. Each local step evaluates the quantum circuit and its gradient, which dominates the per-round computation. KWA incurs O(Nd) memory for storing parameter vectors and $O(N^{2})$ for the temporary affinity matrix. It introduces no additional trainable parameters to the VQC model.

### 3.4. Algorithm

Algorithm 1 summarizes the KWA procedure. The inputs are the *N* local parameter vectors received by the server at a single communication round. The output is the aggregated global parameter vector.

The QFL training loop with KWA follows the structure described in Section 2: for each round *t*, clients perform local optimization via Equations (1) and (2), upload their parameters, and the server invokes Algorithm 1 to produce $\theta^{\left(t+1\right)}$. The only modification to the standard QFL pipeline is replacing the FedAvg averaging step, Equation (4), with the KWA operator, Equation (10).

## 4. Results

In this section, we evaluate KWA on five binary MNIST digit-pair classification tasks under IID, Dir(0.5), and Dir(0.1) data distributions. We primarily compare under IID and Dir(0.5) distributions, while Dir(0.1) serves as a stronger-heterogeneity benchmark. In addition to KWA, we compare the six state-of-the-art QFL methods reviewed in Section 2 and five classical robust aggregation baselines, resulting in twelve methods in total. The classical baselines are coordinate-wise median, coordinate-wise trimmed mean, geometric median, Krum, and Multi-Krum. For N=4 clients and trim level b=1, coordinate-wise median and trimmed mean coincide. We keep both rows for completeness. All experiments use the PennyLane simulator [35] as the quantum backend. The VQC architecture and training protocol match Section 2. Each client trains a ZZFeatureMap with circular entanglement, q=4 qubits, and d=8 parameters. Training runs for T=10 rounds with N=4 clients, S=30 local steps, and Adam [30] at learning rate 0.1. Weighted uses $\alpha_{g}=0.8$ and $\alpha_{\ell }=0.2$. Clustered uses K=2. Density uses $K_{\mathrm{GMM}}=3$. Krum and Multi-Krum use f=1. Multi-Krum averages the m=2 vectors with the smallest Krum scores. The results are averaged over five random seeds (42, 123, 456, 789, 1024). Each reported value is the mean test accuracy with standard deviation across seeds. In the main tables, bold indicates the best QFL value in each column, while underlined values indicate the best classical value.

For paired comparisons, we report a two-sided paired *t*-test and a Wilcoxon signed-rank test on the five seed pairs of each dataset. We also report the 95% bootstrap confidence interval for the mean paired difference and Cohen’s $d_{z}$. We treat pooled tests over all 25 pairs as exploratory, since the five datasets are not independent.

**Figure 2:**
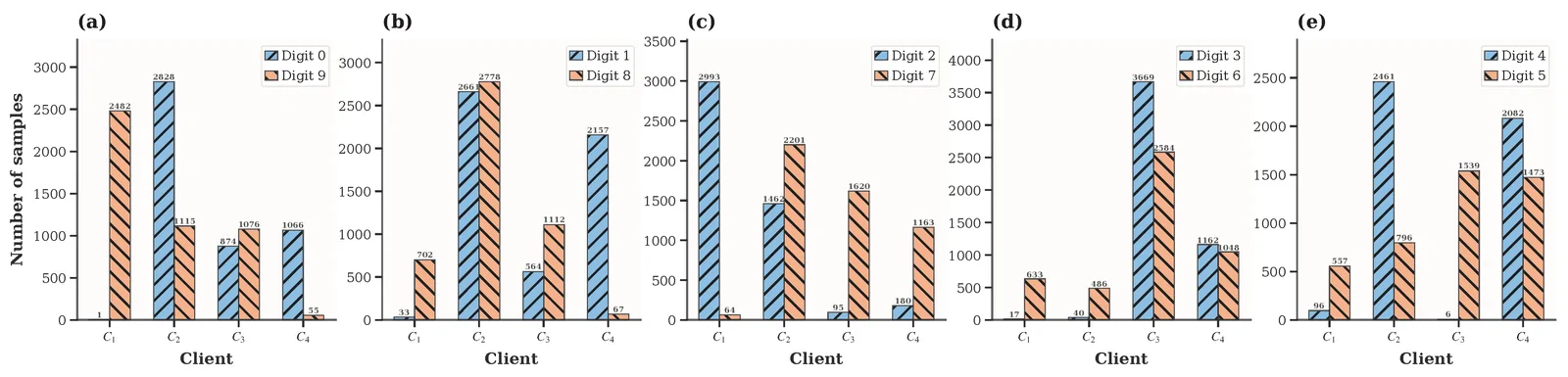
Per-client data distribution under Dir(0.5) with N=4 clients for one representative seed. (**a**) 0 vs. 9, (**b**) 1 vs. 8, (**c**) 2 vs. 7, (**d**) 3 vs. 6, and (**e**) 4 vs. 5. Bars with // hatching indicate the first digit; bars with ∖∖ hatching indicate the second digit. Counts above bars report the number of samples per client.

The main experiments use N=4 clients, the setting adopted by the six reference methods in their original evaluations. Appendix C extends the comparison to N∈{8,16}. Figure 2 illustrates the per-client sample distribution under Dir(0.5) for all five digit pairs (seed 42). One or two clients receive a heavily skewed share of a single class while the remaining clients hold more balanced mixtures. This heterogeneity is the source of client drift that aggregation methods must address.

Table 2 reports the IID results. Among the classical baselines, Krum attains the highest average accuracy (0.7906), whereas among the QFL methods KWA attains the highest average accuracy (0.7881) and FedAvg [18] follows at 0.7870. The mean KWA-FedAvg paired difference is +0.0011, with a pooled paired test p=0.178 and a bootstrap 95% confidence interval of [−0.0002,0.0027] for the mean paired difference. This agreement confirms the theoretical prediction in Section 3: under IID data, kernel weights approach 1/N and KWA reduces to FedAvg.

The five classical baselines attain averages between 0.7890 and 0.7906. Paired tests identify three small but significant differences, all on 4v5: CoordMedian and TrimmedMean exceed KWA by 0.0009 (*t*-test p=0.003), and Multi-Krum exceeds KWA by 0.0013 (*t*-test p=0.028); the Wilcoxon *p*-value for each of the three comparisons is 0.0625. The remaining 22 method-dataset comparisons show no significant difference at the 0.05 level. Weighted falls to 0.7609 and shows high seed-to-seed variability on 2v7 (0.7934±0.151), while Personalized ranks last at 0.5850. No single method dominates every task: Chain leads on 2v7 (0.8714), Chain and Clustered tie on 3v6 (0.8121), and Krum leads on 0v9, 1v8, and 4v5. These isolated wins do not extend to consistent superiority, and KWA and FedAvg remain within a narrow accuracy band across all five tasks.

Table 3 reports the results under Dir(0.5), where non-IID data partitioning introduces client drift. KWA attains the highest average accuracy among all twelve methods (0.7120), FedAvg attains 0.7093, and the KWA-FedAvg margin is +0.0026. Statistical evidence at N=4 is mixed: paired tests on individual datasets give p>0.05 on 0v9 (p=0.544), 2v7 (p=0.128), 3v6 (p=0.897), and 4v5 (p=0.850), whereas FedAvg outperforms KWA on 1v8 by +0.0211 with p=0.00026. The pooled test over 25 pairs gives p=0.763, and the bootstrap 95% confidence interval for the mean paired difference is [−0.012,0.021], which contains zero. We therefore describe KWA at N=4 as comparable to FedAvg with a tendency toward improvement. The client scaling experiment in Appendix C provides the main positive evidence, where the KWA advantage grows to +0.023 at N=16.

The five classical robust baselines rank below FedAvg on average: CoordMedian and TrimmedMean attain 0.6868, GeoMedian attains 0.6823, Multi-Krum attains 0.6464, and Krum attains 0.6324. In pooled paired tests, KWA exceeds Krum by +0.0796 (paired *t*-test p<0.001; Wilcoxon p=0.0001) and Multi-Krum by +0.0655 (paired *t*-test p<0.001; Wilcoxon p=0.0012). KWA also exceeds CoordMedian and TrimmedMean by +0.0251 (paired *t*-test p=0.046; Wilcoxon p=0.067) and GeoMedian by +0.0297 (paired *t*-test p=0.112; Wilcoxon p=0.113). These pooled tests are exploratory, and per-dataset evidence is less uniform: on 2v7, CoordMedian attains 0.7798 while KWA attains 0.7433, and on 4v5, GeoMedian attains 0.7158 while KWA attains 0.6649. Under Dir(0.5), the classical robust baselines do not outperform FedAvg and therefore do not replace it as the primary baseline in the unified protocol.

Among the QFL methods, Weighted, Chain, Clustered, Density, and Personalized attain 0.6702, 0.6417, 0.6180, 0.6040, and 0.5748, respectively, and each ranks below FedAvg. The gap between FedAvg and Weighted is 0.0391, about 15 times the KWA-FedAvg margin. KWA and FedAvg degrade least from their IID levels, by 0.0761 and 0.0777.

We next examine stronger heterogeneity with Dir(0.1), and Table 4 reports the results. KWA attains a mean accuracy of 0.6273, while FedAvg attains 0.5991, with a pooled paired *t*-test p=0.068 and a bootstrap 95% confidence interval of [0.004,0.060] for the mean paired difference. Per-dataset differences do not reach significance, and seed-to-seed variance is large. Density attains the highest average accuracy (0.6296), slightly above KWA. We interpret these results as favorable but not statistically robust evidence for KWA under Dir(0.1). The stronger heterogeneity lowers the accuracies of most methods relative to Dir(0.5); Density is the exception, rising from 0.6040 to 0.6296.

Two additional experiments test generality. Appendix D reports a larger circuit with q=6 qubits and d=12 parameters, where KWA and FedAvg are indistinguishable under IID data but KWA is lower by 0.0528 on 2v7 under Dir(0.5) (paired *t*-test p=0.148). Appendix E reports a three-class MNIST task, where KWA and FedAvg agree within 0.0004 under IID data, but KWA is lower by 0.0197 under Dir(0.5) (paired *t*-test p=0.121). These experiments show that the advantage observed at q=4 does not transfer consistently to larger or multi-class settings.

**Figure 3:**
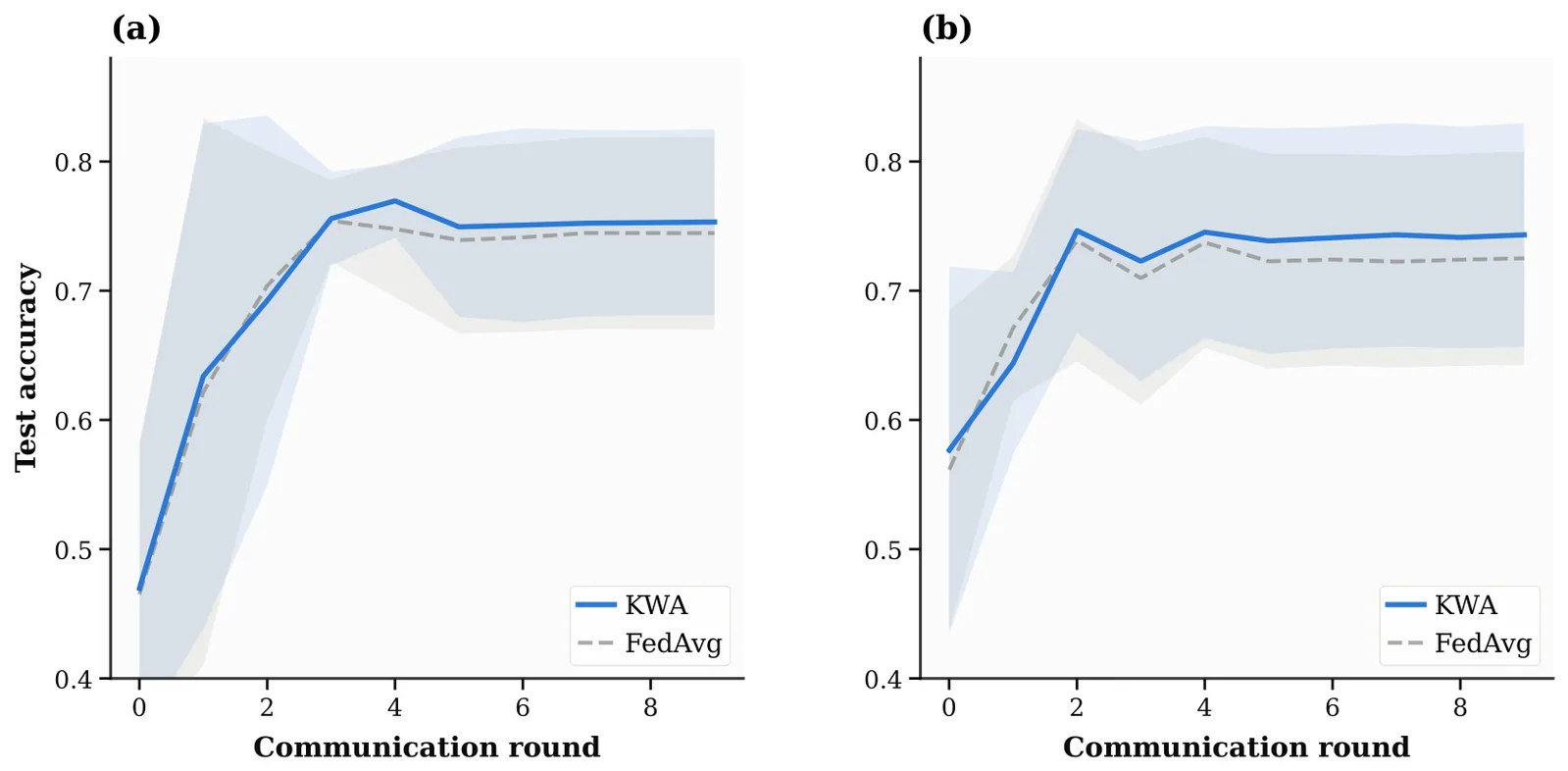
Convergence curves of KWA and FedAvg under Dir(0.5). (**a**) 0 vs. 9 and (**b**) 2 vs. 7. Lines show the mean test accuracy across five seeds (solid: KWA; dashed: FedAvg); shaded regions indicate ± one standard deviation.

**Figure 4:**
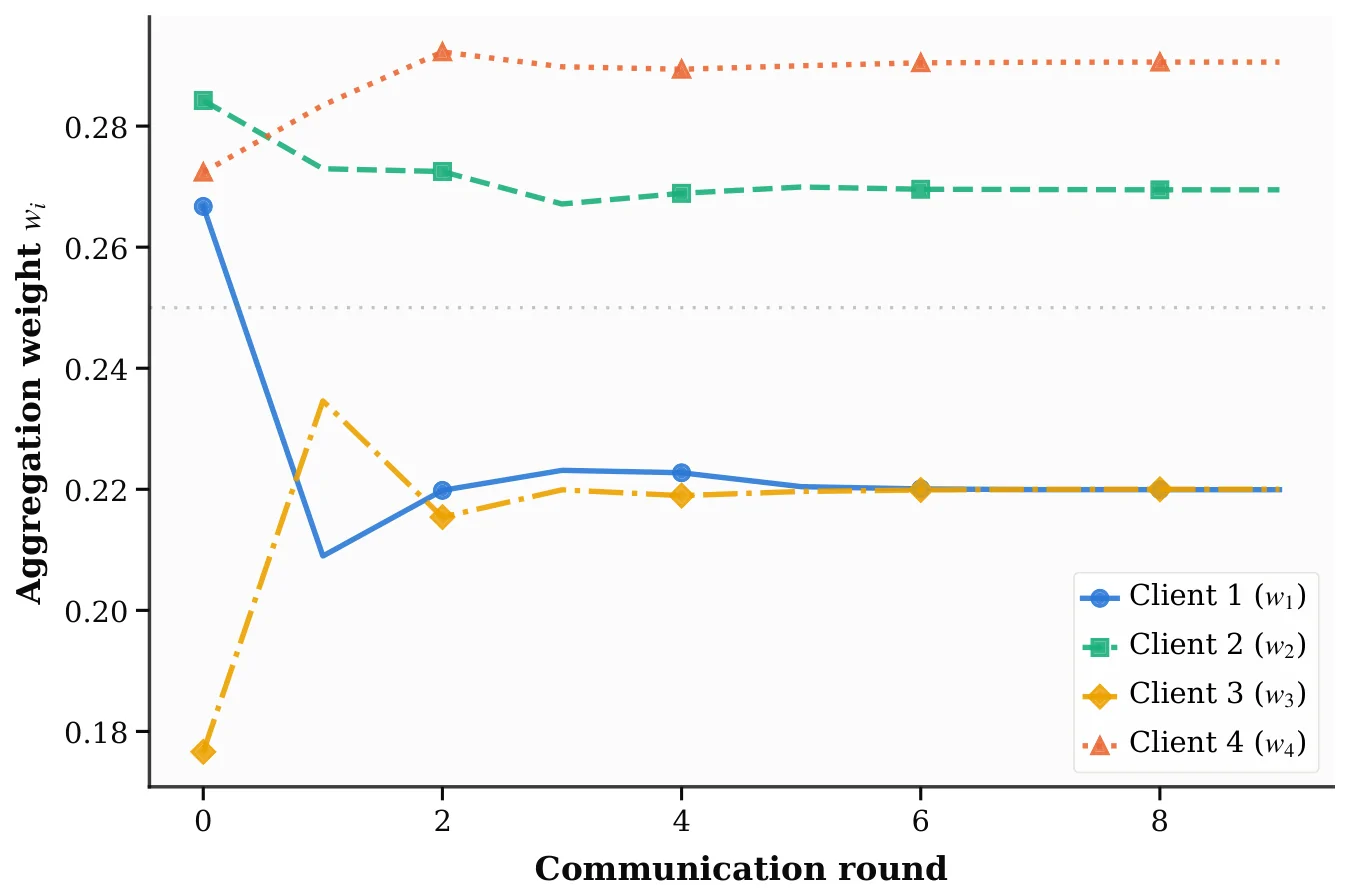
Evolution of KWA aggregation weights $w_{i}$ for N=4 clients across T=10 communication rounds on the 2v7 dataset under Dir(0.5) for one representative seed. Weights adjust modestly in the first two to three rounds and then stabilize, with the final distribution spanning approximately 0.220 to 0.291. Solid/circle: Client 1; dashed/square: Client 2; dash-dot/diamond: Client 3; dotted/triangle: Client 4. The dashed horizontal line marks the uniform baseline $w_{i}=0.25$.

Figure 3 compares the convergence behavior of KWA and FedAvg [18] on the 0v9 and 2v7 datasets under Dir(0.5). Both methods start from similar accuracy in round 0, and as training proceeds the KWA curve separates and maintains a higher accuracy through the final round. This separation coincides with the modest weight adjustment shown in Figure 4.

Figure 4 illustrates how KWA redistributes aggregation weights during training. On a representative Dir(0.5) run on 2v7, the per-client weights $w_{i}$ are tracked across all T=10 communication rounds. In round 0, the weights reflect the initial random parameter positions, and over the first two to three rounds they adjust and then stabilize. The final distribution spans from 0.220 to 0.291, a total spread of approximately 0.07 around the uniform baseline of 0.25. The redistribution is modest under Dir(0.5) but is obtained automatically through the six-step KWA procedure.

**Figure A1:**
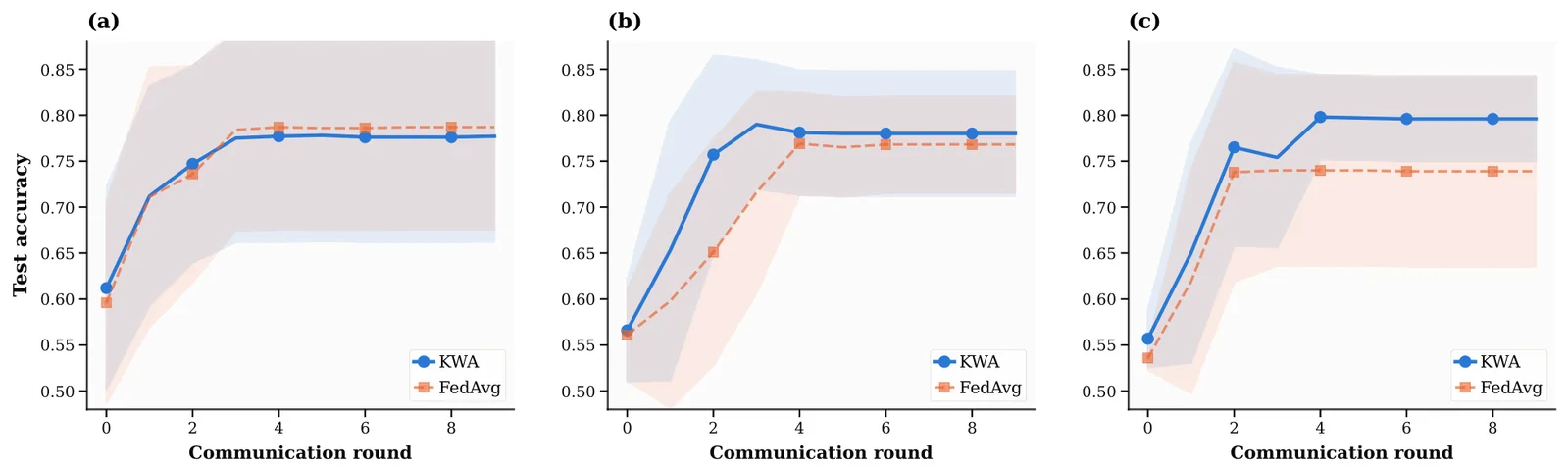
Convergence of KWA and FedAvg [18] under Dir(0.5) on the 2v7 dataset for three federation sizes. (**a**) N=4, (**b**) N=8, and (**c**) N=16. Lines show mean test accuracy across five seeds (solid/circle: KWA; dashed/square: FedAvg); shaded regions indicate ± one standard deviation.

Convergence trajectories at each federation size are shown in Figure A1 of Appendix C, where FedAvg plateaus earlier as *N* increases while KWA continues to improve. The ablation studies in Appendix A and Appendix B provide a further perspective on the KWA design: the bandwidth ablation confirms that data-driven bandwidth selection is essential, and the kernel ablation demonstrates that performance is insensitive to the choice of radial kernel. These results establish KWA as a hyperparameter-free aggregation strategy that ranks first among the twelve methods under Dir(0.5) in average accuracy, is comparable to FedAvg under IID conditions, and requires no manually tuned hyperparameters.

## 5. Discussion and Conclusions

In this work, we have introduced Kernel-Weighted Aggregation (KWA), a hyperparameter-free aggregation strategy for quantum federated learning. KWA treats the local parameter vectors received by the server at each communication round as samples from an unknown distribution, constructs a non-parametric kernel density estimate, and assigns per-client weights proportional to local centrality so that clients in high-density regions receive higher voting power while isolated clients are attenuated. The Gaussian kernel bandwidth is set to the median of the pairwise squared distances, a data-driven heuristic that adapts round by round to the parameter-space geometry. KWA therefore has no manually tuned hyperparameters.

A key contribution of this work is the unified benchmark under which twelve aggregation methods were evaluated. The state-of-the-art QFL methods were originally validated under distinct VQC architectures, qubit counts, optimizers, and datasets; we re-implemented them together with five classical robust baselines under one protocol. All methods use the same PennyLane simulator, the same ZZFeatureMap with q=4 qubits, the same Adam optimizer at learning rate 0.1, and the same five MNIST digit-pair tasks. Under IID data, KWA and FedAvg perform indistinguishably (0.7881 versus 0.7870, Δ=+0.0011), while Krum attains the highest IID average (0.7906) and the top eight methods lie within 0.0061 of one another. Under Dir(0.5) data, KWA attains the highest average accuracy (0.7120), with FedAvg at 0.7093 and the five classical baselines between 0.6324 and 0.6868. Under Dir(0.5), the classical baselines do not outperform FedAvg and therefore do not replace it as the primary baseline in the unified protocol.

The KWA advantage over FedAvg requires careful interpretation. At N=4 clients, the margin under Dir(0.5) is +0.0026, with a pooled paired test p=0.763 and a bootstrap 95% confidence interval of [−0.012,0.021]; we therefore describe KWA as comparable to FedAvg at N=4, with a tendency toward improvement. On the 1v8 task, FedAvg outperforms KWA by +0.0211 with p=0.00026. The client scaling experiment in Appendix C provides stronger evidence: the KWA advantage grows to +0.023 at N=16, driven mainly by 2v7 where FedAvg degrades from 0.787 to 0.739 while KWA rises to 0.796. We characterize KWA by three structural properties: it is hyperparameter-free, it adaptively down-weights isolated clients through data-driven centrality weighting, and its advantage scales favorably with federation size.

KWA is not a Byzantine-robust aggregator: it targets client drift under statistical heterogeneity among honest clients and is not designed to resist coordinated malicious clients, since an adversary can manipulate the pairwise distances and the median bandwidth. We therefore do not claim robustness against Byzantine attacks. The classical robust baselines Krum [31] and median-based rules [32] provide explicit Byzantine tolerance under their assumptions, but under Dir(0.5) they did not outperform FedAvg. Future work may combine KWA with Byzantine-robust selection rules.

The practical significance of hyperparameter-free operation merits emphasis. Weighted requires two blending coefficients, Clustered requires *K*, and Density requires $K_{\mathrm{GMM}}$, while the optimal value of each hyperparameter depends on the unknown degree of non-IID severity. KWA removes this burden: its bandwidth is determined from the data at each round by the median heuristic. The bandwidth ablation in Appendix A confirms that the median heuristic does not require precise calibration, and the kernel ablation in Appendix B shows that Gaussian, Laplace, and Cauchy kernels all agree within 0.004. These results are consistent with prior work on data-driven bandwidth selection for kernel density estimation [33,34].

Several limitations define directions for future work. The main evaluation is confined to binary MNIST digit pairs with q=4 qubits and d=8 parameters, and the larger-circuit experiment in Appendix D shows mixed results under Dir(0.5), while the three-class experiment in Appendix E shows KWA below FedAvg by 0.0197. These experiments indicate that the advantage observed at q=4 does not transfer consistently to larger or multi-class settings. Under Dir(0.1), KWA attains 0.6273 and FedAvg attains 0.5991, a favorable but not statistically robust trend with p=0.068, so stronger heterogeneity and multi-class tasks require further study. Future work could combine KWA with proximal regularization [24] or control variates [25] to improve drift correction.

In conclusion, this research establishes Kernel-Weighted Aggregation as a principled hyperparameter-free alternative to FedAvg for quantum federated learning under non-IID data. The method has no manually tuned hyperparameters, adapts automatically to the client parameter geometry, and recovers FedAvg in the IID limit. Under Dir(0.5) data, KWA attains the highest average accuracy among twelve methods, with a small margin over FedAvg at N=4 that becomes substantial at N=16 in the client scaling experiment. The unified benchmark also shows that existing QFL aggregation methods do not consistently outperform FedAvg under controlled comparison, underlining the importance of standardized experimental protocols in quantum federated learning research [22]. The processed datasets and experimental configurations required to reproduce all results are publicly available in an AtomGit repository at https://atomgit.com/RuiHuang/KWA (accessed on 24 August 2026).

## Figures and Tables

**Table 1:** Summary of existing QFL aggregation methods. All methods operate on *d*-dimensional parameter vectors from *N* clients. The limitation column highlights the primary vulnerability relevant to the small-client regime (N=4).

Method	Hyperparameters	Output	Primary Limitation
FedAvg [18]	None	Global	Uniform weighting under drift
Weighted [26]	$\alpha_{g},\alpha_{\ell }$	Per-client	Coefficients not adaptive
Personalized [26]	None	Per-client	Hard threshold; memory cost
Chain [27]	None	Global	Order dependency; error cascade
Clustered [28]	*K*	Global	Non-deterministic; *K* selection
Density [29]	$K_{\mathrm{GMM}}$	Global	GMM fit with small Nd

**Table 2:** Test accuracy of twelve aggregation methods under IID data. The first seven rows are QFL methods and the last five rows are classical robust baselines. Per-dataset values report the mean and standard deviation across five seeds. The Avg column reports the pooled mean and standard deviation over the 25 per-seed observations. Bold indicates the best QFL value in each column, while underline indicates the best classical value.

Method	0v9	1v8	2v7	3v6	4v5	Avg
KWA	0.8098±0.009	0.6928±0.017	0.8700±0.008	0.8109±0.009	0.7569±0.010	0.7881±0.061
FedAvg [18]	0.8092±0.008	0.6871±0.019	0.8704±0.008	0.8115±0.008	0.7568±0.009	0.7870±0.063
Chain [27]	0.8091±0.009	0.6734±0.018	0.8714±0.010	0.8121±0.008	0.7564±0.008	0.7845±0.067
Clustered [28]	0.8084±0.008	0.6682±0.047	0.8693±0.008	0.8121±0.009	0.7581±0.008	0.7832±0.071
Density [29]	0.8087±0.009	0.6616±0.053	0.8664±0.009	0.8118±0.008	0.7576±0.009	0.7812±0.073
Weighted [26]	0.8093±0.009	0.6331±0.073	0.7934±0.151	0.8115±0.008	0.7569±0.009	0.7609±0.101
Personalized [26]	0.5720±0.223	0.5351±0.168	0.5762±0.227	0.6570±0.112	0.5846±0.105	0.5850±0.180
CoordMedian	0.8097±0.009	0.6969±0.016	0.8699±0.009	0.8110±0.009	0.7577±0.009	0.7890±0.059
TrimmedMean	0.8097±0.009	0.6969±0.016	0.8699±0.009	0.8110±0.009	0.7577±0.009	0.7890±0.059
GeoMedian	0.8098±0.009	0.6978±0.022	0.8710±0.007	0.8109±0.009	0.7577±0.010	0.7894±0.060
Krum	0.8103±0.009	0.7082±0.022	0.8676±0.012	0.8084±0.010	0.7584±0.009	0.7906±0.055
Multi-Krum	0.8103±0.008	0.7034±0.022	0.8682±0.011	0.8095±0.009	0.7582±0.010	0.7899±0.057

**Table 3:** Test accuracy of twelve aggregation methods under Dir(0.5). The first seven rows are QFL methods and the last five rows are classical robust baselines. Per-dataset values report the mean and standard deviation across five seeds. The Avg column reports the pooled mean and standard deviation over the 25 per-seed observations. Bold indicates the best QFL value in each column, while underline indicates the best classical value.

Method	0v9	1v8	2v7	3v6	4v5	Avg
KWA	0.7533±0.072	0.7106±0.006	0.7433±0.086	0.6877±0.104	0.6649±0.081	0.7120±0.084
FedAvg [18]	0.7446±0.075	0.7317±0.006	0.7252±0.082	0.6822±0.118	0.6631±0.074	0.7093±0.086
Weighted [26]	0.6514±0.140	0.6938±0.070	0.6757±0.050	0.6501±0.119	0.6797±0.049	0.6702±0.095
Chain [27]	0.6324±0.159	0.5214±0.113	0.7019±0.126	0.7078±0.115	0.6450±0.137	0.6417±0.147
Clustered [28]	0.6973±0.094	0.6958±0.078	0.6252±0.090	0.5352±0.047	0.5366±0.061	0.6180±0.105
Density [29]	0.5602±0.106	0.6535±0.055	0.6019±0.097	0.5411±0.086	0.6636±0.101	0.6040±0.103
Personalized [26]	0.6083±0.191	0.5921±0.146	0.5391±0.175	0.5676±0.049	0.5670±0.104	0.5748±0.144
CoordMedian	0.7496±0.088	0.6523±0.027	0.7798±0.094	0.6214±0.113	0.6311±0.093	0.6868±0.109
TrimmedMean	0.7496±0.088	0.6523±0.027	0.7798±0.094	0.6214±0.113	0.6311±0.093	0.6868±0.109
GeoMedian	0.7688±0.055	0.6220±0.037	0.7067±0.133	0.5980±0.132	0.7158±0.074	0.6823±0.114
Krum	0.6539±0.090	0.5996±0.043	0.6685±0.139	0.5989±0.138	0.6409±0.137	0.6324±0.119
Multi-Krum	0.6854±0.101	0.5737±0.039	0.7126±0.127	0.6078±0.148	0.6526±0.144	0.6464±0.129

**Table 4:** Test accuracy of the seven QFL methods under Dir(0.1). Per-dataset values report the mean and standard deviation across five seeds. The Avg column reports the pooled mean and standard deviation over the 25 per-seed observations, and the best results are in bold.

Method	0v9	1v8	2v7	3v6	4v5	Avg
KWA	0.7311±0.136	0.6974±0.076	0.5308±0.176	0.6530±0.147	0.5239±0.036	0.6273±0.152
FedAvg [18]	0.6557±0.149	0.6880±0.101	0.5234±0.169	0.5996±0.122	0.5287±0.042	0.5991±0.141
Weighted [26]	0.6280±0.119	0.5974±0.122	0.5167±0.154	0.5697±0.108	0.5013±0.019	0.5626±0.124
Personalized [26]	0.5785±0.144	0.5320±0.136	0.5268±0.172	0.5606±0.079	0.4957±0.048	0.5387±0.128
Clustered [28]	0.6701±0.102	0.6033±0.113	0.5798±0.187	0.6398±0.129	0.4852±0.020	0.5956±0.138
Chain [27]	0.6855±0.108	0.6630±0.027	0.6638±0.163	0.5197±0.059	0.4894±0.018	0.6043±0.124
Density [29]	0.7253±0.148	0.5991±0.050	0.6348±0.110	0.6124±0.148	0.5766±0.105	0.6296±0.129

## Data Availability

The processed datasets, along with the experimental configurations required to reproduce the results, are available in a public AtomGit repository at https://atomgit.com/RuiHuang/KWA (accessed on 24 August 2026).

## Appendix A. Bandwidth Ablation

We evaluate four bandwidth variants of KWA on two representative datasets (0v9 and 2v7) subsampled to 1000 samples under IID and Dir(0.5) distributions with five seeds. The four variants are: (i) KWA (median bandwidth, the proposed default); (ii) KWA-mean (bandwidth set to the mean of squared pairwise distances); (iii) KWA-$h^{2}=0.01$ (fixed small bandwidth); and (iv) KWA-$h^{2}=1.0$ (fixed large bandwidth, approximating FedAvg). Table A1 and Table A2 report the results.

**Table A1:** Test accuracy of bandwidth variants under IID data. Mean accuracy with standard deviation across five seeds, and the best results are in bold.

Method	0v9	2v7	Avg
KWA (median)	0.7540±0.027	0.8210±0.023	0.7875±0.042
KWA-mean	0.7530±0.026	0.8200±0.023	0.7865±0.041
KWA-$h^{2}=1.0$	0.7500±0.024	0.8210±0.022	0.7855±0.043
KWA-$h^{2}=0.01$	0.6630±0.114	0.7600±0.137	0.7115±0.135

**Table A2:** Test accuracy of bandwidth variants under Dir(0.5). Mean accuracy with standard deviation across five seeds, and the best results are in bold.

Method	0v9	2v7	Avg
KWA (median)	0.7670±0.033	0.7770±0.115	0.7720±0.085
KWA-mean	0.7680±0.030	0.7850±0.112	0.7765±0.082
KWA-$h^{2}=1.0$	0.7530±0.055	0.7830±0.116	0.7680±0.092
KWA-$h^{2}=0.01$	0.6030±0.107	0.6210±0.105	0.6120±0.106

Table A1 and Table A2 report the four variants, which span three bandwidth regimes. Replacing the median by the mean changes the average accuracy by 0.001 under IID and by 0.0045 under Dir(0.5). This confirms that the median heuristic does not require precise calibration. A fixed large bandwidth ($h^{2}=1.0$) slightly degrades performance, consistent with the behavior of an overly broad kernel that approaches uniform weights and loses adaptivity to parameter-space structure. A fixed small bandwidth ($h^{2}=0.01$) causes a large accuracy loss: the kernel collapses to near-zero affinities between most client pairs, centrality scores become numerically unstable, and accuracy drops by 0.06 to 0.16 across both distributions. These results validate the necessity of data-driven bandwidth selection. The median heuristic is sufficient and introduces no tuning burden.

## Appendix B. Kernel Function Ablation

We compare three kernel functions for computing pairwise affinities: (i) Gaussian $\exp(-d_{ij}^{2}/2h^{2})$, the proposed default; (ii) Laplace $\exp(-d_{ij}/h)$ with scale parameter $h=\sqrt{\mathrm{median}(d_{ij}^{2})}$; and (iii) Cauchy $1/(1+d_{ij}^{2}/h^{2})$ with the same $h^{2}$ as Gaussian. The experimental protocol matches Appendix A.

Table A3 reveals that kernel function choice has negligible impact on KWA performance. Under IID data, the Laplace kernel holds a marginal advantage with an average accuracy of 0.7890, followed by the Gaussian kernel at 0.7875 and the Cauchy kernel at 0.7870. The maximum pairwise difference across all three kernels is 0.0020 in this regime. Under Dir(0.5), the ranking reverses: the Gaussian kernel leads at 0.7720, the Cauchy kernel follows at 0.7705, and the Laplace kernel trails at 0.7685. The spread is even narrower here, with a maximum difference of 0.0035. Averaging over both distributions, no kernel outperforms another by more than 0.004, a margin smaller than the typical within-method standard deviation. This insensitivity to kernel specification constitutes a practical strength. Unlike methods that introduce hyperparameters requiring per-dataset tuning, KWA achieves consistent performance with the three radial kernels tested. The Gaussian kernel is retained as the default for its smoothness, its theoretical connection to the squared Euclidean distance metric used in Equation (5), and its widespread adoption in kernel methods.

**Table A3:** Test accuracy of kernel function variants on two datasets with 1000 samples. Mean accuracy with standard deviation across five seeds, and the best results are in bold.

Method	IID			Dir(0.5)		
	0v9	2v7	Avg	0v9	2v7	Avg
Gaussian	0.7540±0.027	0.8210±0.023	0.7875±0.042	0.7670±0.033	0.7770±0.115	0.7720±0.085
Laplace	0.7550±0.028	0.8230±0.024	0.7890±0.043	0.7640±0.037	0.7730±0.121	0.7685±0.089
Cauchy	0.7530±0.026	0.8210±0.023	0.7870±0.042	0.7660±0.034	0.7750±0.114	0.7705±0.084

## Appendix C. Client Scaling

We examine how the KWA advantage over FedAvg scales with the number of clients. Experiments are conducted on 0v9 and 2v7 (1000 samples each) with N∈{4,8,16} clients, IID and Dir(0.5) distributions, and five seeds. Table A4 and Table A5 report the results.

Table A4 and Table A5 report the scaling behavior of KWA and FedAvg across N∈{4,8,16} clients. Under IID data, both methods perform comparably across all client counts, with KWA maintaining a marginal advantage of +0.0015 to +0.0025 per *N*. The Δ row in Table A5 reveals the key pattern under Dir(0.5). At N=4, the two methods are nearly tied with an average difference of +0.0005. At N=8, KWA develops a modest lead of +0.007. At N=16, KWA achieves a substantial advantage of +0.023, driven primarily by a +0.057 gain on the 2v7 dataset where FedAvg degrades sharply from 0.787 at N=4 to 0.739 at N=16. This scaling behavior is consistent with the design principle of KWA: as more clients join the federation, the parameter-space density estimation benefits from a larger sample, and the distinction between consensus and outlier regions sharpens. FedAvg, by contrast, treats every client equally and becomes increasingly vulnerable to the accumulation of client-specific drift as *N* grows.

**Table A4:** Test accuracy of FedAvg and KWA under IID data with N∈{4,8,16} clients. Mean accuracy with standard deviation across five seeds, and the best results are in bold.

Method	N=4		N=8		N=16	
	0v9	2v7	0v9	2v7	0v9	2v7
FedAvg [18]	0.750±0.024	0.820±0.023	0.752±0.022	0.819±0.023	0.749±0.024	0.819±0.021
KWA	0.754±0.027	0.821±0.023	0.751±0.022	0.823±0.021	0.749±0.021	0.823±0.022

**Table A5:** Test accuracy of FedAvg and KWA under Dir(0.5) with N∈{4,8,16} clients. Mean accuracy with standard deviation across five seeds; Δ denotes the KWA advantage over FedAvg for each (N,dataset) pair, and the best results are in bold.

Method	N=4		N=8		N=16	
	0v9	2v7	0v9	2v7	0v9	2v7
FedAvg [18]	0.756±0.056	0.787±0.111	0.760±0.032	0.768±0.052	0.777±0.025	0.739±0.104
KWA	0.767±0.033	0.777±0.115	0.762±0.030	0.780±0.068	0.766±0.028	0.796±0.046
Δ (KWA − FedAvg)	+0.011	−0.010	+0.002	+0.012	−0.011	+0.057

Figure A1 visualizes this trend through convergence curves of KWA and FedAvg on the 2v7 dataset under Dir(0.5) for each federation size. At N=4, both methods follow similar trajectories, with FedAvg holding a marginal final advantage (0.787 versus 0.777). At N=8, KWA begins to separate: it converges faster in early rounds and maintains a lead through round 10 (0.780 versus 0.768, Δ=+0.012). At N=16, the divergence becomes pronounced: FedAvg plateaus near 0.74 after round 2, while KWA continues to improve, reaching 0.796 at round 10 (Δ=+0.057). This progressive widening of the convergence gap shows that centrality-based weighting sharpens its discrimination as more parameter vectors become available for density estimation.

## Appendix D. Larger-Circuit Experiment

We examine whether KWA remains effective when the VQC grows from q=4, d=8 to q=6, d=12, using the same data pipeline as the main benchmark except that PCA retains six components. We use two representative digit pairs, 0v9 and 2v7, with five seeds, N=4 clients, and the same Adam settings; Table A6 reports the results.

**Table A6:** Test accuracy of KWA and FedAvg with q=6 qubits and d=12 parameters. Mean accuracy with standard deviation across five seeds, and the best results are in bold.

Method	0v9 IID	0v9 Dir(0.5)	2v7 IID	2v7 Dir(0.5)
FedAvg	0.6045±0.008	0.6608±0.044	0.7993±0.013	0.8416±0.034
KWA	0.6045±0.008	0.6569±0.054	0.7994±0.013	0.7888±0.064

Under IID data, KWA and FedAvg are indistinguishable on both tasks. Under Dir(0.5), KWA is lower by 0.0039 on 0v9 (paired *t*-test p=0.716) and by 0.0528 on 2v7 (paired *t*-test p=0.148, Wilcoxon p=0.0625). These results show that the advantage observed at q=4 does not transfer consistently to the larger circuit.

## Appendix E. Three-Class Classification Experiment

We examine whether KWA extends to multi-class classification on the MNIST digits 0, 3, and 6. The VQC uses q=4 qubits and d=8 parameters; the first three *Z* expectations provide three logits, and training uses softmax cross-entropy. The protocol keeps N=4 clients, five seeds, ten rounds, and Adam at learning rate 0.1; Table A7 reports the results.

**Table A7:** Test accuracy of KWA and FedAvg on the three-class MNIST task 0/3/6. Mean accuracy with standard deviation across five seeds, and the best results are in bold.

Method	IID	Dir(0.5)
FedAvg	0.7448±0.033	0.6659±0.101
KWA	0.7443±0.034	0.6462±0.105

Under IID data, the two methods agree within 0.0004, while under Dir(0.5), KWA is lower by 0.0197 (paired *t*-test p=0.121). The three-class experiment therefore shows no KWA advantage under moderate heterogeneity, but it confirms that the KWA aggregation operator can be inserted into a multi-class QFL loop without modifying the VQC training protocol.
